# In-stent thrombosis after 68 months of implantation inspite of continuous dual antiplatelet therapy: a case report

**DOI:** 10.1186/1757-1626-3-68

**Published:** 2010-02-23

**Authors:** Tarun Nagrani, Medhat Zaher, Sainath Gaddam, George Jabbour, Duccio Baldari, Roberto Baglini, Srinivas Duvvuri

**Affiliations:** 1 Department of Medicine, Staten Island University Hospital, 475 Seaview Ave, Staten Island, NY-10305, USA; 2 Department of Cardiology, Staten Island University Hospital, 475 Seaview Ave, Staten Island, NY-10305, USA; 3 Department of Cardiology, Istituto Mediterraneo per I Trapianti e le Terapie ad Alta Specializzazione (ISMETT), University of Pittsburgh Medical Center in Italy

## Abstract

Lately, there has been an increased incidence of late stent thrombosis; especially following Drug eluting stent (DES) implantation. Several factors are associated with an increased risk of stent thrombosis, including the procedure itself, patient and lesion characteristics, stent design, and premature cessation of anti-platelet drugs. We present a case of late stent thrombosis (LST) following DES implantation after a period of 68 months, making it the longest reported case of LST reported in the literature, despite the use of dual anti-platelet therapy.

## Introduction

Several randomized clinical trials have shown that implanting Drug eluting stents (DES) result in clinically significant reductions of in-stent re-stenosis compared with Bare metal stents (BMS) [1,2]. However increased risk of Late Stent thrombosis (LST) is more pronounced 6 months to 1 year after DES implantation, compared with BMS [3-5]. The benefits of lower rate of in-stent re-stenosis versus the risk of in-stent thrombosis is paramount in the decision making process especially when faced with a patient requiring emergency stenting. In-stent thrombosis is a much rarer event than in-stent re-stenosis but results in mortality rate of up to 45% and a nonfatal infarction rate of another 30% to 40% [6,7]. Our patient experienced very late stent thrombosis, occurring 68 months post DES implantation, making it the longest time-to-event, reported in the literature.

## Case presentation

A 75-year-old Caucasian gentleman had presented to our emergency department 5 years and 8 months ago (2003) with chest pain secondary to anterior wall ST-segment elevation myocardial infarction. His past medical history was significant for hypertension and dyslipidemia with a 50 pack years smoking history. Dual oral anti-platelet therapy with aspirin and clopidogrel along with intravenous heparin were administered. Emergent left heart catheterization and selective coronary angiography revealed thrombotic 90% occlusion of the mid left anterior descending coronary artery. Balloon angioplasty and thombectomy were performed. A sirolimus eluting stent (3.0 × 33) was placed at maximum pressure of 10 atmospheres across the lesion. Angiography after stent deployment showed complete expansion of the stent with grade 3 TIMI flow, without proximal or distal edge dissections or any residual stenosis. The patient received intravenous abciximab bolus and infusion therapy for 12 hours and had an uneventful hospital course.

Subsequently, the patient had been strictly compliant with dual antiplatelet therapy of aspirin and clopidogrel.

**Figure 1:**
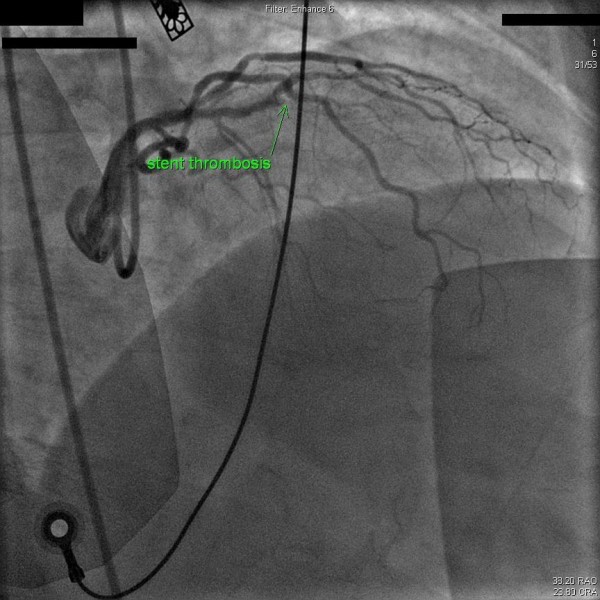
**Angiographic evidence of in-stent thrombosis in the mid-segment of previous LAD stent**.

**Figure 2:**
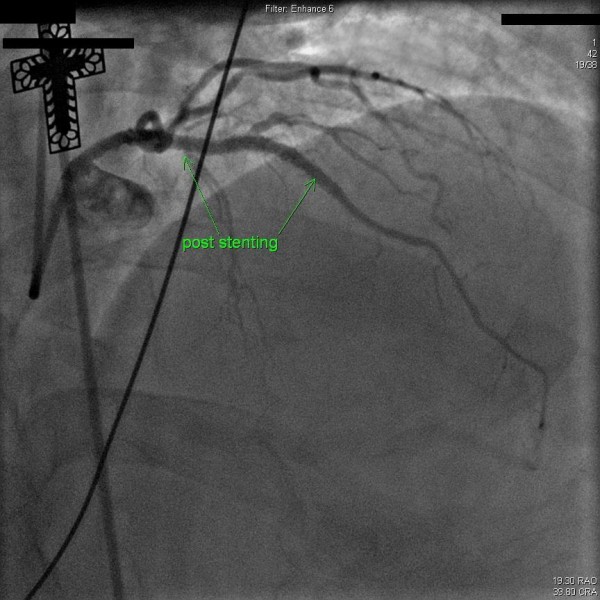
**Angiographic evidence of blood flow (TIMI-3) in the mid-LAD segment post stenting**.

However about three weeks prior to this admission (2009), he had two mild episodes of angina on exertion. A persantine-technetium 99 myocardial perfusion imaging stress test carried out was interpreted as positive for anterior wall reversible ischemia. Few days later, he presented to our hospital with sudden onset chest tightness and an EKG showing ST-segment elevation in leads V1-2. Emergent coronary angiography revealed a filling defect with 100% occlusion in the mid segment of the previous LAD stent with TIMI-0 flow, consistent with in-stent thrombosis along with significant proximal in-stent restenosis (Figure 1). Intravenous bivalirudin and eptifibatide were administered. Balloon angioplasty was performed at the in-stent thrombosis site. An Everolimus RX stent (3.0 × 15) was placed across the lesion resulting in 0% residual stenosis. Another Everolimus RX stent (3.0 × 23) was deployed at the proximal edge of the previous stent and a smaller Everolimus RX stent (3.0 × 8) was deployed in an 80% de-novo lesion leaving no residual stenosis (Figure 2). Peak plasma troponin level was 60.05 ng/ml. A transthoracic echocardiogram performed after 48 hours showed mild hypokinesis of anterior wall of the left ventricle, an estimated ejection fraction of 45%. After 3 days of hospitalization patient was discharged on daily medications of aspirin 325 mg daily and clopidogrel 75 mg daily, atorvastatin 80 mg, isosorbide mononitrate 30 mg daily, atenolol 50 mg daily and lisinopril 20 mg daily.

## Discussion

Stent thrombosis (ST) is classified by Academic Research Consortium into four types based on the time of event as; Acute: within 24 hours, Subacute: 24 hours to 30 days, Late: after 30 days, and very late: after 12 months. The consequences of ST could be catastrophic with a mortality rate of 45%, with the majority of others suffering nonfatal myocardial infarction [6,7]. Angioscopic and optical coherence tomography studies have shown that lack of complete endothelialization of DES to be the most important predictor of LST [8,9]. Virmani et al have confirmed the same mechanism using human pathological data [10]. Risk factors for LST include patient and lesion characteristics, non-compliance of anti-platelet drugs and procedural factors like incomplete stent apposition, number and the length of stents, coronary dissection, etc. [11]. It has been reported that the risk of stent thrombosis up to 4 years is significantly higher if the DES was placed during ACS [12]. In the setting of myocardial infarction, underlying plaque morphology may affect the rate of healing, when stent struts penetrate deeply into a necrotic core and are not in contact with cellular areas, which impairs stent endothelialization [13]. A large thrombus burden in the setting of ST-segment elevation myocardial infarction is a risk factor for future stent thrombosis [14]. Hypercholesterolemia has recently been shown to cause endothelial progenitor cell dysfunction, thus contributing to the delay in endothelial healing, adding to the significance of statin therapy after PCI [15]. There is consensus among cardiologists that stent endothelialization starts from the edges towards the center of the stent, leaving the middle portion of the stent to be the last to be covered by endothelium and usually the most likely site for LST [10]. The same authors reported LST to be associated with diffuse in stent re-stenosis. The following factors could have contributed to the development of in-stent thrombosis in our patient: long stent [33 mm in length], history of hypercholesterolemia, implantation of DES emergently during ST-segment myocardial infarction with high thrombus burden necessitating rheolytic thrombectomy and the presence of in-stent re-stenosis.

The 2007 ACC/AHA/SCAI focused update of the 2005 guidelines on PCI recommended dual antiplatelet therapy with aspirin and a theinopyridine for at least 12 months after DES implantation. Recent reports of very late in-stent thrombosis of DES long years after implantation represent a growing evidence to continue dual antiplatelet therapy for a longer period of time, perhaps indefinitely given the devastating consequences of in-stent thrombosis. A retrospective observational trial has suggested that triple antiplatelet therapy using aspirin, clopidogrel and cilostazol may further reduce the risk of stent thrombosis especially in patients or lesions at increased risk with no difference in the rate of major bleeding [16]. The advent of prasugrel as a more potent platelet inhibitor compared to clopidrogrel may decrease the long term adverse cardiovascular events [17]. Finally, this case highlights the need of further long-term studies on the occurrence of very late in-stent restenosis in patients treated with DES, both as an independent factor, and as predisposing to very late stent thrombosis.

## Abbreviations

ACS: acute coronary syndrome; BMS: bare metal stent; DES: drug eluting stent; LST: late stent thrombosis; PCI: per cutaneous intervention.

## Competing interests

The authors declare that they have no competing interests.

## Authors' contributions

TN is the major contributor to the case presentation of the manuscript and conducted literature review. MZ is the major contributor to the Discussion section of the manuscript and was involved in direct patient care of the patient. GJ and SG were involved in direct patient care. DB and RB were the reviewing cardiologists. SD is the interventional cardiologist directly involved in patient care.

## Consent

Written informed consent was obtained from the patient's next of kin/family for publication of this case report. A copy of the written consent is available for review by the Editor-in-Chief of this journal.

## References

[B1] Moses JW Leon MB Popma JJ Fitzgerald PJ Holmes DR O'Shaughnessy C Caputo RP Kereiakes DJ Williams DO Teirstein PS Jaeger JL Kuntz RE Sirolimus-eluting stents versus standard stents in patients with stenosis in a native coronary artery N Engl J Med 2003 349 1315 1323 10.1056/NEJMoa035071 14523139

[B2] Stone GW Ellis SG Cox DA Hermiller J O'Shaughnessy C Mann JT Turco M Caputo R Bergin P Greenberg J Popma JJ Russell ME A polymer-based, paclitaxel-eluting stent in patients with coronary artery disease N Engl J Med 2004 350 221 231 10.1056/NEJMoa032441 14724301

[B3] Daemen J Wenaweser P Tsuchida K Abrecht L Vaina S Morger C Kukreja N Jüni P Sianos G Hellige G van Domburg RT Hess OM Boersma E Meier B Windecker S Serruys PW Early and late coronary stent thrombosis of sirolimus-eluting and paclitaxel-eluting stents in routine clinical practice: data from a large two-institutional cohort study Lancet 2007 369 667 678 10.1016/S0140-6736(07)60314-6 17321312

[B4] Lagerqvist B James SK Stenestrand U Lindback J Nilsson T Wallentin L Long-term outcomes with drug-eluting stents versus bare-metal stents in Sweden N Engl J Med 2007 356 1009 1019 10.1056/NEJMoa067722 17296822

[B5] Stone GW Moses JW Ellis SG Schofer J Dawkins KD Morice MC Colombo A Schampaert E Grube E Kirtane AJ Cutlip DE Fahy M Pocock SJ Mehran R Leon MB Safety and efficacy of sirolimus- and paclitaxel-eluting coronary stents N Engl J Med 2007 356 998 1008 10.1056/NEJMoa067193 17296824

[B6] Iakovou I Schmidt T Bonizzoni E Ge L Sangiorigi GM Stankovic G Airoldi F Chieffo A Montorfano M Carlino M Michev I Corvaja N Briguori C Gerckens U Grube E Colombo A Incidence, predictors, and outcome of thrombosis after successful implantation of drug-eluting stents JAMA 2005 293 2126 2130 10.1001/jama.293.17.2126 15870416

[B7] Pfisterer M Brunner-La Rocca HP Buser PT Rickenbacher P Hunziker P Mueller C Jeger R Bader F Osswald S Kaiser C Late clinical events after clopidogrel discontinuation may limit the benefit of drug-eluting stents: an observational study of drug-eluting versus bare-metal stents J Am Coll Cardiol 2006 48 2584 2591 10.1016/j.jacc.2006.10.026 17174201

[B8] Kotani J Awata M Nanto S Uematsu M Oshima F Minamiguchi H Mintz GS Nagata S Incomplete neointimal coverage of sirolimus-eluting stents angioscopic findings J Am Coll Cardiol 2006 47 10 2108 11 10.1016/j.jacc.2005.11.092 16697331

[B9] Matsumoto D Shite J Shinke T Otake H Tanino Y Tanino Y Ogasawara D Sawada T Paredes OL Hirata K Yokoyama M Neointimal coverage of sirolimus-eluting stents at 6-month follow-up: evaluated by optical coherence tomography Eur Heart J 2007 28 8 961 967 10.1093/eurheartj/ehl413 17135281

[B10] Finn AV Joner M Nakazawa G Kolodgie F Newell J John MC Gold HK Virmani R Pathological Correlates of Late Drug-Eluting Stent Thrombosis: Strut Coverage as a Marker of Endothelialization Circulation 2007 115 2435 2441 10.1161/CIRCULATIONAHA.107.693739 17438147

[B11] Luscher TF Steffel J Eberli FR Joner M Nakazawa G Tanner FC Virmani R Drug-eluting stent and coronary thrombosis: biological mechanisms and clinical implications Circulation 2007 115 8 1051 1058 10.1161/CIRCULATIONAHA.106.675934 17325255

[B12] Wenaweser P Daemen J Zwahlen M van Domburg R Jüni P Vaina S Hellige G Tsuchida K Morger C Boersma E Kukreja N Meier B Serruys PW Windecker S Incidence and Correlates of Drug-Eluting Stent Thrombosis in Routine Clinical Practice: 4-Year Results From a Large 2 Institutional Cohort Study J Am Coll Cardiol 2008 52 1134 1140 10.1016/j.jacc.2008.07.006 18804739

[B13] Farb A Burke AP Kolodgie FD Virmani R Pathological Mechanisms of Fatal Late Coronary Stent Thrombosis in Humans Circulation 2003 108 1701 1706 10.1161/01.CIR.0000091115.05480.B0 14504181

[B14] Sianos G Papafaklis MI Daemen J Vaina S van Mieghem CA van Domburg RT Michalis LK Serruys PW Angiographic stent thrombosis after routine use of drug-eluting stents in ST-segment elevation myocardial infarction: the importance of thrombus burden J Am Coll Cardiol 2007 50 573 583 10.1016/j.jacc.2007.04.059 17692740

[B15] Pirro M Bagaglia F Paoletti L Razzi R Manarino MR Review: Hypercholesterolemia-associated progenitor cell dysfunction Ther Adv Cardiovasc Dis 2008 2 5 329 339 10.1177/1753944708094769 19124431

[B16] Singh I Shafiq N Pandhi P Reddy S Pattanaik S Sharma Y Malhotra S Triple antiplatelet therapy vs. dual antiplatelet therapy in patients undergoing percutaneous coronary intervention: an evidence-based approach to answering a clinical query Br J Clin Parmacol 2009 68 1 4 13 10.1111/j.1365-2125.2009.03402.x PMC2732935 19659998

[B17] Unger EF Weighing benefits and risks--FDA's review of prasugrel N Engl J Med 2009 361 10 942 945 10.1056/NEJMp0907122 19726770

